# Serum RNA Profiling in the 10-Years Period Prior to Diagnosis of Testicular Germ Cell Tumor

**DOI:** 10.3389/fonc.2020.574977

**Published:** 2020-10-28

**Authors:** Joshua Burton, Sinan U. Umu, Hilde Langseth, Tom Grotmol, Tom K. Grimsrud, Trine B. Haugen, Trine B. Rounge

**Affiliations:** ^1^Department of Lifesciences and Health, OsloMet - Oslo Metropolitan University, Oslo, Norway; ^2^Department of Research, Cancer Registry of Norway, Oslo, Norway; ^3^Department of Informatics, University of Oslo, Oslo, Norway

**Keywords:** RNA profiling, serum, testicular cancer, pre-diagnostic, seminoma, non-seminoma, sequencing, miRNA

## Abstract

Although testicular germ cell tumor (TGCT) overall is highly curable, patients may experience late effects after treatment. An increased understanding of the mechanisms behind the development of TGCT may pave the way for better outcome for patients. To elucidate molecular changes prior to TGCT diagnosis we sequenced small RNAs in serum from 69 patients who were later diagnosed with TGCT and 111 matched controls. The deep RNA profiles, with on average 18 million sequences per sample, comprised of nine classes of RNA, including microRNA. We found that circulating RNA signals differed significantly between cases and controls regardless of time to diagnosis. Different levels of TSIX related to X-chromosome inactivation and TEX101 involved in spermatozoa production are among the interesting findings. The RNA signals differed between seminoma and non-seminoma TGCT subtypes, with seminoma cases showing lower levels of RNAs and non-seminoma cases showing higher levels of RNAs, compared with controls. The differentially expressed RNAs were typically associated with cancer related pathways. Our results indicate that circulating RNA profiles change during TGCT development according to histology and may be useful for early detection of this tumor type.

## Introduction

Testicular germ cell tumor (TGCT) is diagnosed in 1% of men worldwide and is the most common malignancy in males between 20 and 39 years of age. The incidence rates are still rising (1), and some of the highest incidences are found in Northern Europe (2). The etiology is largely unknown, although genetic components and conditions during pregnancy seem to play a role (3). The susceptibility to TGCT is shown to have a strong familial link, with a fourfold increased risk for fathers and eightfold for brothers (4, 5). The polygenic nature of TGCT has been recognized and more than 50 susceptibility genes have been identified (6–9). The susceptibility loci contain genes linked to germ cell development and sex determination, as well as genes related to tumor growth/suppression. Cryptorchidism is a well-known risk factor, and around 10% of males with TGCT have a history of cryptorchidism (10). The main histologic subtypes of TGCT are seminoma, non-seminoma and mixed, making up around 50, 30, and 20%, respectively (11).

TGCT is a highly curable disease since the introduction of cisplatin-based chemotherapy with a 5-year survival rate of above 95% (12, 13). However, there is an increased risk of long-term side effects including secondary non-germ cell (GC) cancers, cardiovascular disease, and hypogonadism (14–16).

The current guidelines presented by the European Association of Urology (17) for the diagnosis of TGCT include several techniques, including ultrasound imaging for initial diagnosis and serum markers for specific subtype diagnosis and prognosis (18–20). The serum markers used are alpha-fetoprotein, hCG and LDH, however alpha-fetoprotein is only seen in yolk-sac tumor and teratomas whilst hCG is expressed by trophoblasts only (21). Whilst LDH is a less specific serum marker, the serum level is usually proportional to tumor volume and levels may be increased in advanced TGCT (17). The presence of histological markers is also used for diagnosis. For germ cell neoplasia *in situ* (GCNIS), the markers include OCT3/4, PLAP, CD117, and SALL 4 (17). Furthermore, it has been shown that the metabolic biomarkers leptin and resisting may predict cancer mortality due to their function of inducing pro-tumorigenic environment that further promotes tumor initiation, angiogenesis, and metastasis (22).

RNAs, such as microRNA (miRNA), piwi-interacting RNA (piRNA), and long non-coding RNA (lncRNA), regulate gene expression on transcriptional and post-transcriptional levels and have been shown to be present in serum (23). The differential composition of RNAs in circulation can help determine diagnosis as well as the developmental stage of the tumor (24). Circulating miRNAs have been identified as both prognostic and diagnostic markers in biliary tract cancer (BTC), which has led to earlier diagnosis and less invasive procedure compared with previously used techniques (25). Exosomal miRNAs have also been observed in colorectal cancer (CRC). Specifically, miR-150-5p and miR-99b-5p were found to be downregulated in CRC patients compared to healthy patients (26). Differential levels of circulating RNA were also observed in pre-diagnostic serum samples in lung cancer patients, with an overall dynamic trend when advancing clinically (27).

In TGCT patients, high expression of miRNAs belonging to the clusters *miR-302/367* and *miR-371-373* has been found in serum (28–30). Small RNA sequencing performed on TGCT tissue samples revealed miRNAs profiles to differ between normal and TGCT tissues, as well as between histological subtypes. A genome wide downregulation or loss of piRNAs was observed in TGCT, through mechanisms such as hypermethylation in CpG islands on genes associated with piRNAs (8, 31–33).

Circulating RNAs in TGCT patients have allowed for a less invasive and a more specific histology at diagnosis (17). However, pre-diagnostic circulating RNA changes in TGCT have yet to be studied.

The aim of this study was to investigate the role of RNA in the development of TGCT in serum samples collected in a 10-years period prior to diagnosis. Furthermore, we investigated how differences in circulating RNAs were related to histologies and time periods before diagnosis. Differences in RNAs profiles were then used in functional enrichment analysis, and RNAs with stable patterns were investigated further.

## Materials and Methods

### Study Design and Participants

All samples included were retrieved from the Janus Serum Bank (JSB). JSB is a population-based cancer research biobank containing serum samples from 318 628 individuals collected from 1972 to 2004 (34, 35). The TGCT cases were identified by linking the JSB to the Cancer Registry of Norway using the Norwegian individually unique national identity numbers. The prediagnostic serum samples were donated up to 10 years before the diagnosis. We drew 111 cancer-free Janus participants for comparison of RNA levels with the cancer cases. The control subjects had to be alive and free from cancer, except for non-melanoma skin cancer, at the time of their matched case's diagnosis, and up to 10 years after blood collection. Controls were matched on age, time of blood collection and blood donor group (dependent on their county of residence, year of collection and method of storage), and as a result of these matching criteria, the average age at sample donation was 35 years (standard deviation of 6.5/6.7, respectively) for both cases and controls.

Previous studies have shown the feasibility of using long-term archived serum samples for RNA analyses and the variability of the RNA levels (23, 31, 36). The methods and analyses of the TGCT serum samples were similar to studies by Umu et al. (27). The donors have given broad consent for the use of the samples in cancer research. The study was approved by the Norwegian regional committee for medical and health research ethics (REC no: 24 846, 2012/1590).

### Laboratory Processing of Serum to RNA Profiles

We extracted RNAs from 400 μl serum using phenol-chloroform phase separation and the miRNeasy Serum/Plasma kit (Cat. no 1071073, Qiagen) using a QIAcube (Qiagen). NEBNext® Small RNA Library Prep Set for Illumina (Cat. No E7300, New England Biolabs Inc.) was used for small RNA-seq library preparation. RNA molecules from 17 to 47 nt in length were selected. We sequenced 12 samples per lane of a HiSeq 2500 (Illumina). Additional information is available in our previous study (23).

### Bioinformatics Analyses

Total number of reads generated was 3.5 billion with an average sampling depth of 18.4 million raw reads. AdapterRemoval v2.1.7 (37) was used to trim for adapters. We then mapped the collapsed reads to human genome version hg38 with Bowtie2 v2.2.9, with 10 alignments per read being allowed. Our annotation set consist of miRBase(v.22) (38) for miRNAs, pirBAse for piRNAs (39) and GENCODE (40) for other RNAs. IsomiR and tRF profiles were obtained through SeqBuster (41) and MINTmap, respectively (42). In the analyses, we included RNAs with at least five reads in more than 20% of the samples. Details are available in our previous study (23).

The *optmatch* R package (github.com/markmfredrickson/optmatch) allowed us to find optimally matched sets of controls (Supplementary Table 1) for each analysis. The analyses were matched on age, histology when appropriate and technical artifacts. The technical artifacts accounted for differences in pre-analytical treatment and storage time (23, 31), termed blood donor groups. The DESeq2 R package (v1.18.1) (43) was used for the differential expression analyses using the default generalized linear model with a negative binomial distribution. We consider differences in expression outside the range{−1,1} log_2_ fold change (log_2_fc) as biologically meaningful and are counted in the summary bar charts. Venn diagrams, produced with the R package (VennDiagram, v. 1.6.20), represent common and unique differentially expressed RNA trends among the categories.

Kyoto Encyclopedia of Genes and Genomes (KEGG) pathway analysis was performed using kegga function from the limma R package. The inputs included mRNA, miRNA and isomiR targets extracted from miRDB (v5.0) predictions (44) with a cut off score of >60. With the mRNA and miRNA targets we also performed a pathway visualization (pathview, v.1.22.3) of selected pathway seen in the KEGG enrichment analysis (45, 46).

To exploit the full statistical power of the dataset when identifying RNAs that differ in cases and controls regardless of other factors, we first compared all cases and controls. To identify histology (determined by ICD-O (3rd revision) codes) specific RNA signals, we compared the RNA levels of seminoma with those of the matched controls, and similarly for non-seminomas and matched controls. To identify RNA signals according to the time between blood draw and diagnosis we divided prediagnostic time into four discrete time intervals, 0–2, 2–5, 5–8, and 8–10 years. We selected these intervals to optimize resolution on time to diagnosis while still having sufficient statistical power. To make the time windows comparable with respect to statistical power, the cut points were chosen to secure the same number of cases and controls, and similar proportions of histologies.

## Results

### Reduced Levels of RNAs in TGCT Cases

**Figure 1:**
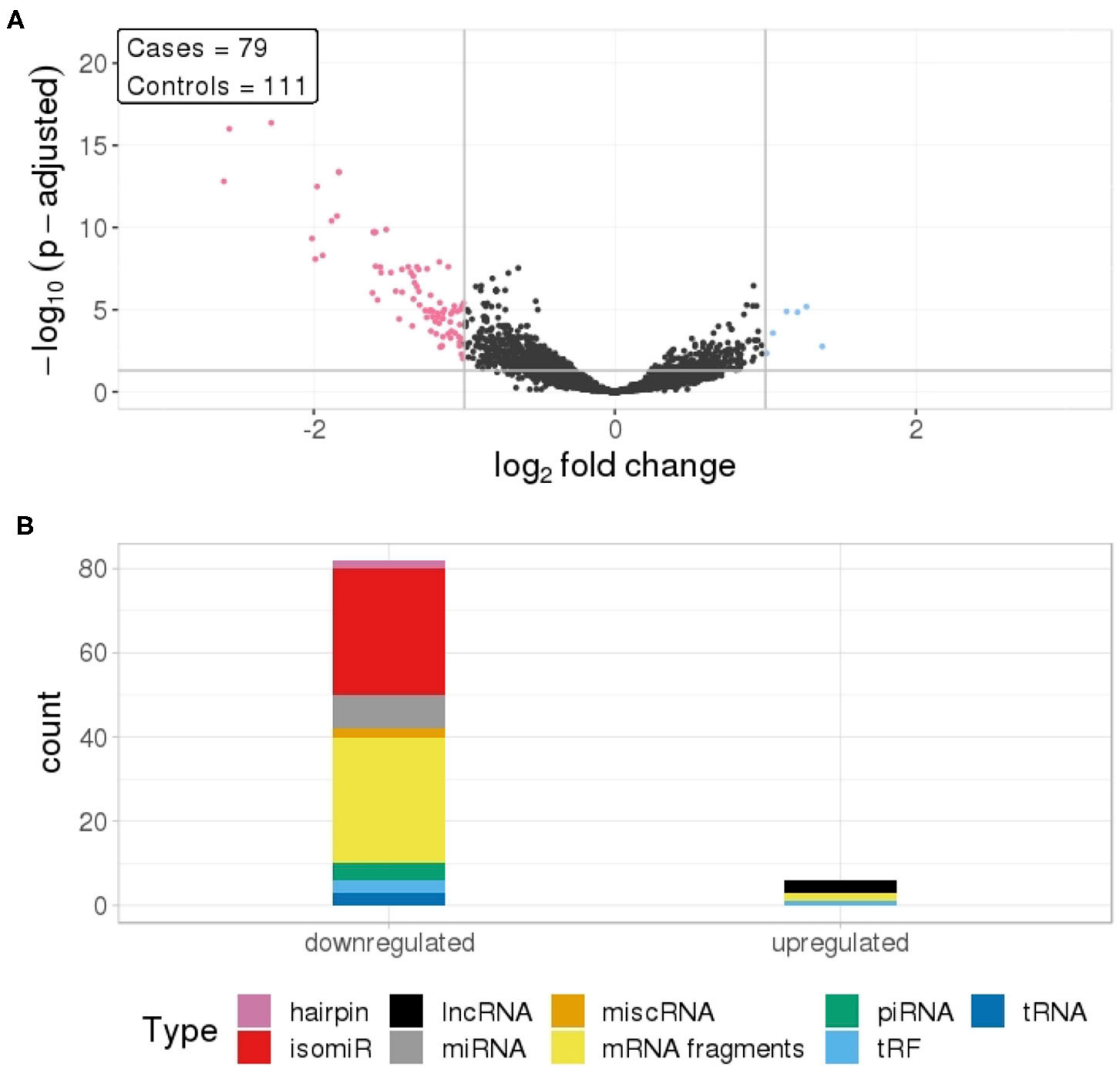
**(A)** Differential RNA levels in pre-diagnostic serum samples from 79 TGCT cases and 111 matched controls. Pink represent RNAs with >1 log_2_ fold change reduced levels in cases and blue represent RNAs with >1 log_2_ fold change increased levels in cases, compared to controls. **(B)** RNA class composition of the significantly elevated and reduced RNAs.

To identify differentially expressed RNAs in all TGCT cases (*n* = 79) against matched controls (*n* = 111), we analyzed 4231 RNAs that passed our inclusion criteria. Of these, we identified 818 RNAs that were differentially expressed with a *p*-adjusted value ≤0.05, and 88 of these had a log_2_ fold change (log_2_fc) outside the range {−1,1} (Figure 1A). The majority of these RNAs (82) had reduced levels in cases compared to controls, with an average log_2_fc = −1.37 and *p*-adjusted = 0.00032. The RNAs showing reduced levels were primarily isomiRs and mRNA fragments, whereas the few elevated RNAs consisted of lncRNAs, mRNA fragments, and a tRF (Figure 1B).

### RNA Signals Differ With Histological Subtype

We compared RNA levels in seminoma and non-seminoma cases with control samples. As seen for all cases combined, seminomas exhibited a similar pattern with reduced level for the majority of the differentially expressed RNAs.

**Figure 2:**
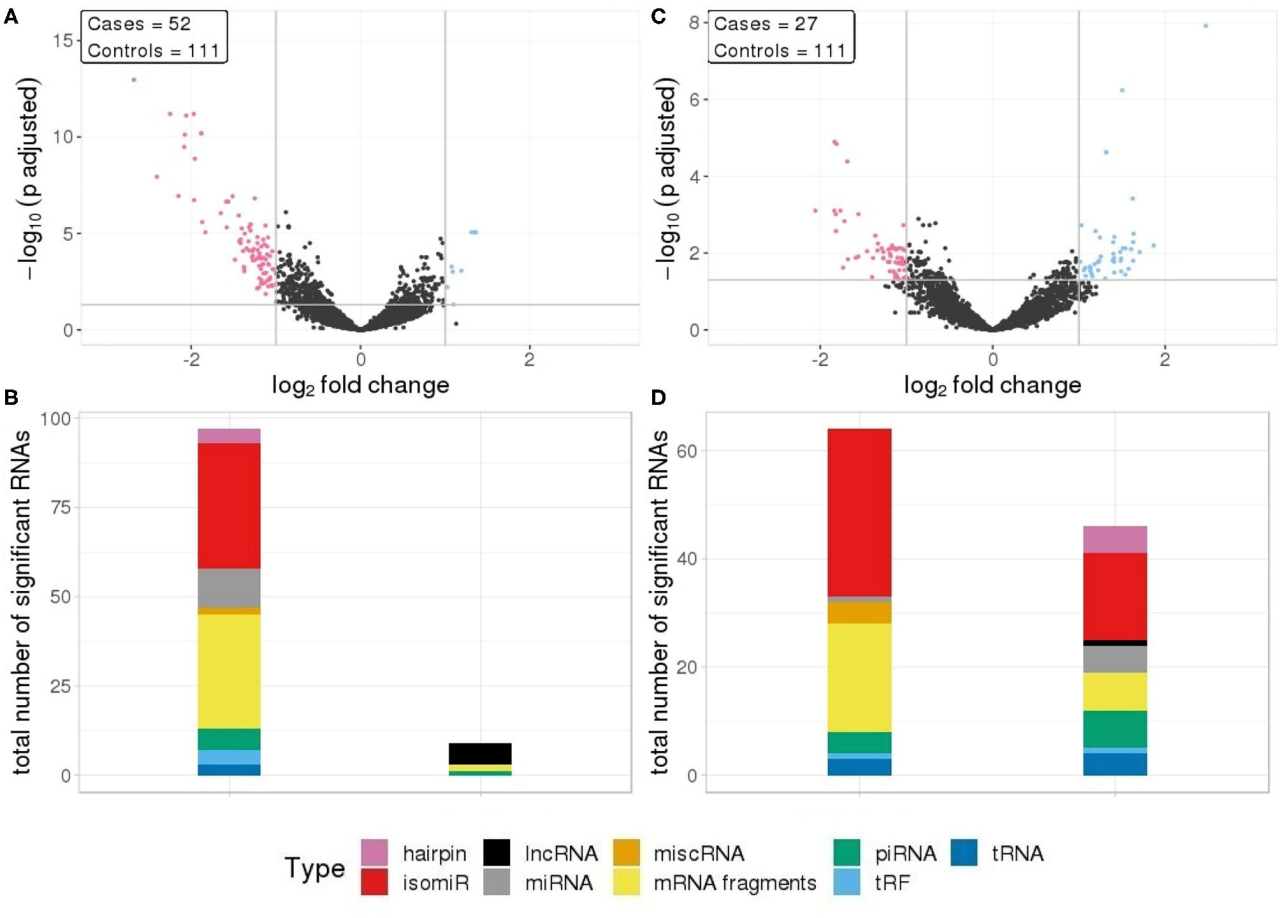
**(A)** Differential RNA expression between seminomas and controls; **(B)** Differential RNA expression between non-seminomas and controls; Pink represent RNAs with >1 log_2_ fold change reduced levels in cases and blue represent RNAs with >1 log_2_ fold change increased levels in cases, compared to controls. **(C,D)** RNA class composition of elevated and reduced RNA levels with associated histology.

In total, 112 RNA signals were different in seminomas compared to controls, 102 with lower RNA levels and 10 with elevated levels (Figure 2A). Similar to all TGCT cases, the majority of the downregulated RNAs, with a mean log_2_fc values of −1.35, consisted of mRNA fragments and isomiRs, whereas the upregulated RNAs consisted of lncRNAs, mRNA fragments, piRNAs, and tRFs (Figure 2B).

In contrast to TGCT and seminomas, where the differences compared to controls were generally large, non-seminomas had 63 RNAs with reduced levels and 46 RNAs with elevated levels (Figure 2C), with mean log_2_fc values of −0.131 and 1.34, respectively. The majority of the RNAs with reduced levels consisted of mRNA fragments and isomiRs, as was also observed with all TGCT, a result driven by the seminomas. However, in non-seminomas the elevated RNAs showed higher proportions of isomiRs, and mRNA fragments, piRNAs and miRNAs as the second most abundant types (Figure 2D).

### RNA Signals Are Not Associated With Time to Diagnosis

**Figure 3:**
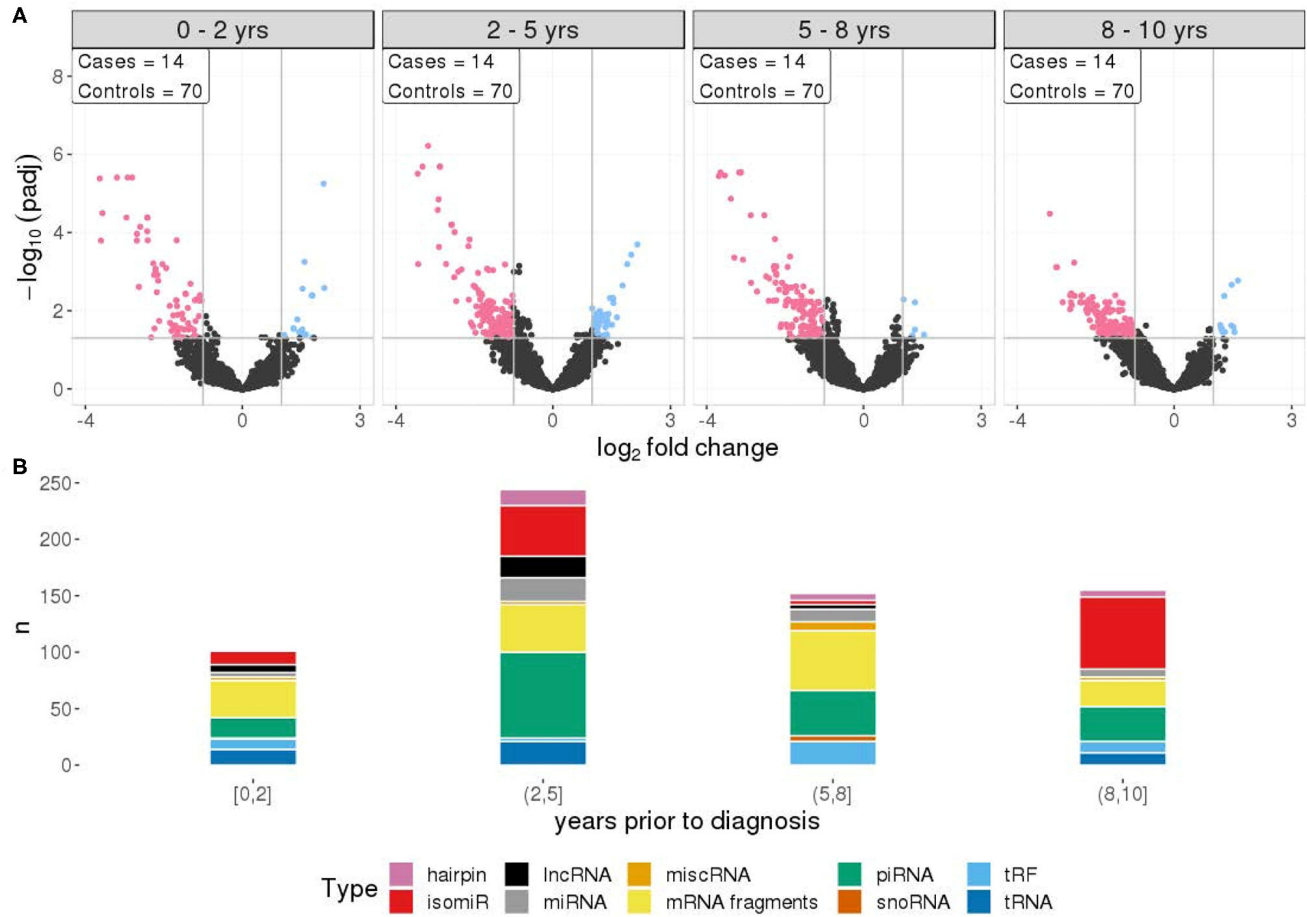
**(A)** Differential RNA expression between cases and matched controls, across four time frames prior to diagnosis with equal sample sizes. Pink represent RNAs with >1 log_2_ fold change reduced levels in cases and blue represent RNAs with >1 log_2_ fold change increased levels in cases, compared to controls. **(B)** RNA class composition of elevated and reduced significant RNA levels for the associated time frames.

To investigate if there is any association between RNA signals and time to diagnosis, four different time frames during the prediagnostic period were examined. The RNA signals in all time frames exhibited similar patterns to the overall prediagnostic TGCT patterns, with the majority of significantly differentially expressed RNAs having reduced levels. This was strongest in the 2–5 years time frame with 282 RNA levels being reduced and 64 RNAs with elevated levels. The 0–2 years time frame showed the least amount of differentially expressed RNAs, with 93 RNAs showing reduced levels and 18 with elevated levels (Figure 3A). Fragments containing mRNAs constitute the major proportion of the significantly changed RNAs in all time frames. However, in the 2–5 years time frame, a higher number of piRNAs (76 out of 244) had different levels, and furthermore, in the 8–10 years, the higher proportion was isomiRs (64 out of 155) (Figure 3B).

### Specific RNA Signals Are Independent of Histology and Time to Diagnosis

**Figure 4:**
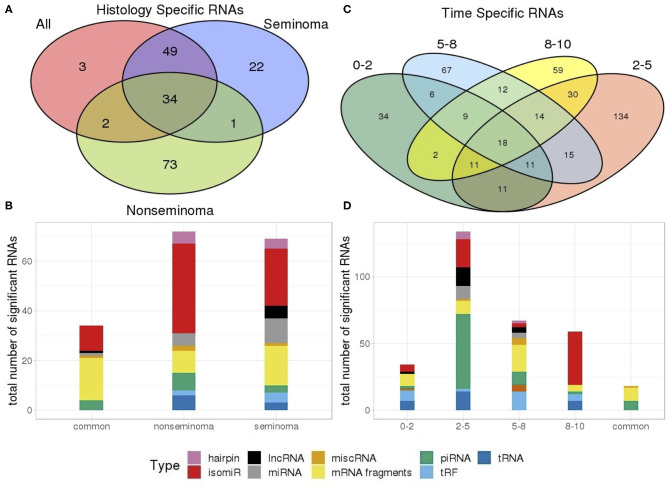
Venn diagram showing the number of **(A)** RNAs which are dependent and independent on histological subtype; **(B)** RNAs which are dependent and independent on single time periods or are observed in multiple time periods. **(C)** Types of RNAs differentially present in only seminomas and non-seminomas, and the 35 common types shared between both; **(D)** Types of differentially present RNAs unique to each time frame as well as those 18 shared between all four time frames.

A number of RNAs are specific or common to histologies and time frames as illustrated by the venn diagrams in Figure 4. A total of 83 out of 88 of the RNAs identified were observed in both seminoma separately and all TGCT profiles. Furthermore, seminomas and non-seminomas shared 35 RNAs, and the majority of these were either mRNA fragments or isomiRs (Figures 4A,B).

An investigation into the common RNAs between time frames showed that 18 RNAs are consistently differentially expressed at all time frames, and that these RNAs were piRNAs and mRNA fragments. RNAs from the 2–5 years time frame, which has the highest number of RNAs above our cut-off, consisted of primarily piRNAs (Figures 4C,D).

### mRNA Fragments Have Consistently Reduced Levels in TGCT Cases Compared to Controls

**Figure 5:**
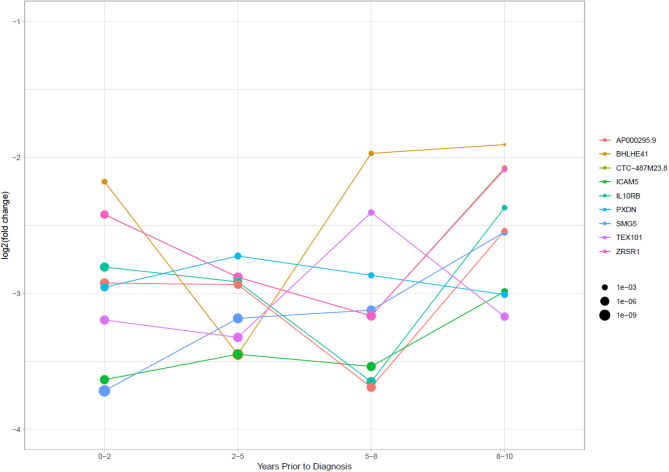
Log_2_ fold change of RNAs that had significantly stably changed levels between cases and controls in all four time periods. Point size is equal to the *p*-adjusted value for the RNAs in each time frame.

To investigate the stability of TGCT RNAs over time, we looked at nine RNAs with the lowest adjusted *p*-values which appeared in all four time frames and their associated log_2_fc. Primarily, the significantly reduced RNAs were mRNA fragments which showed a relatively stable log_2_fc over time. BHLHE41 showed some dynamic changes across the time frames with a log_2_fc range between −1.91 to −3.45, however, this could still be considered stable due to the overall log_2_fc remaining negative. Of all the mRNAs, a slight overall increase in log_2_fc was observed over time, with a mean log_2_fc of −2.91 at 0–2 years and −2.52 at 8–10 years. However, the lowest average log_2_fc appears at 2–5 years with a mean of −3.08, followed by a mean of −3.06 at 5–8 years. This showed that during the extended time frame of 0–8 years, there was a stability in the RNA levels, with a slight increase at 8–10 years (Figure 5).

### Common Cancer Related Pathways Were Enriched in Specific Time Periods and Histologies

Enrichment analysis showed multiple pathways enriched for significant miRNA targets and mRNA fragments. Of the significant pathways, only one [Axon guidance [mean *p*-adjusted = 0.0094]] appeared in all seven subgroups, and two appeared in six of the seven sub-analyses [Ras signaling pathway [mean *p*-adj = 0.10], MAPK signaling pathway [mean *p*-adj = 0.043]]. In total there are 19 pathways that span at least three of the time frames, with the remaining six only appearing in two of the four. Non-seminoma and 0–2 years time frame had the lowest number of significant enriched pathways with only three pathways in each.

**Figure 6:**
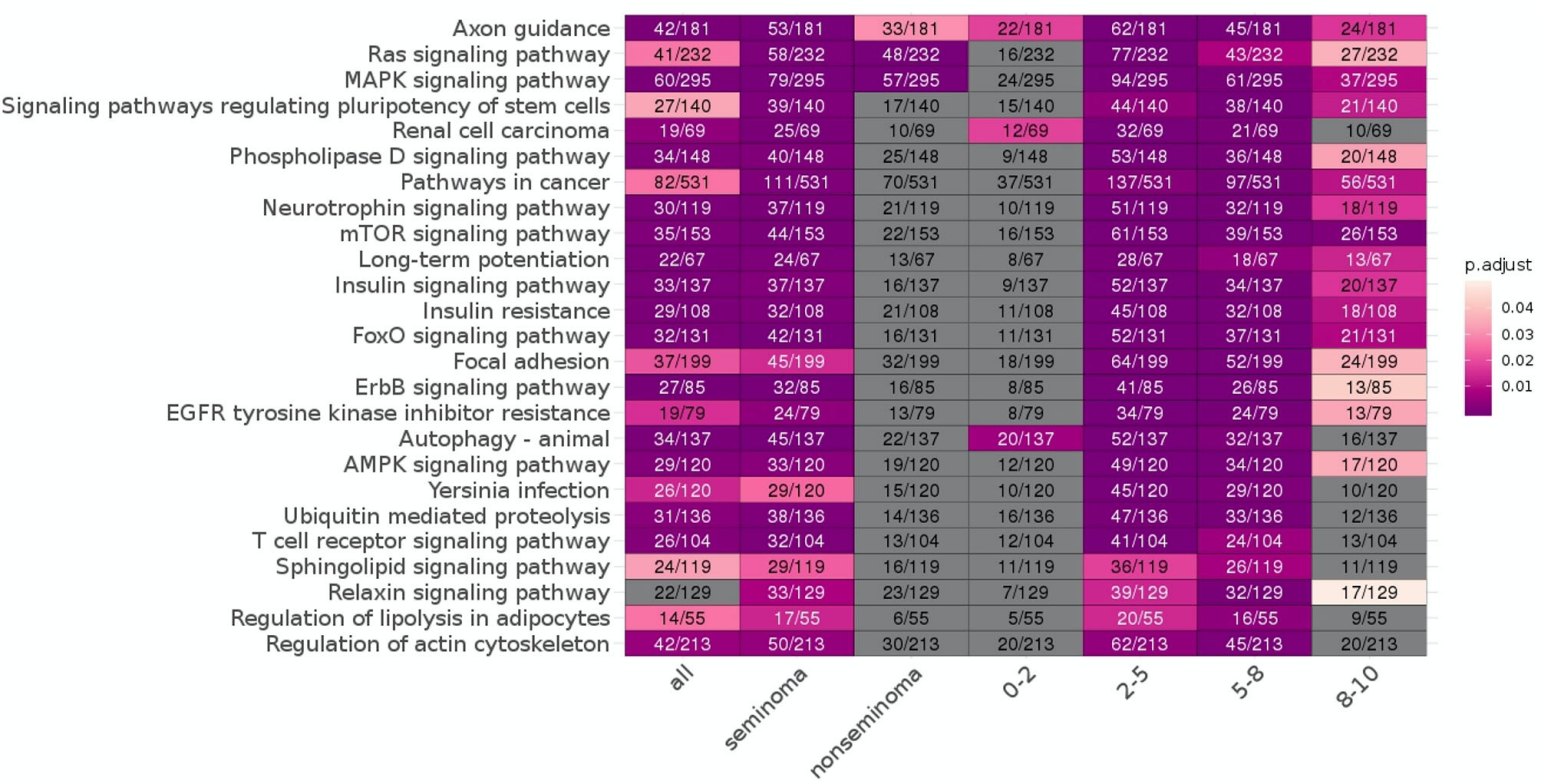
KEGG pathways enriched for differentially expressed mRNAs and miRNA targets for all TGCT vs. controls, seminoma vs. controls, non-seminomas vs. controls, 0–2, 2–5, 5–8, and 8–10 years prior to diagnosis vs. controls are shown. The pathways are color coded according to *p*-values and the number of enriched genes and total number of genes in the pathways are also shown. The 25 most commonly enriched pathways for these analyses were selected for presentational purposes.

**Figure 7:**
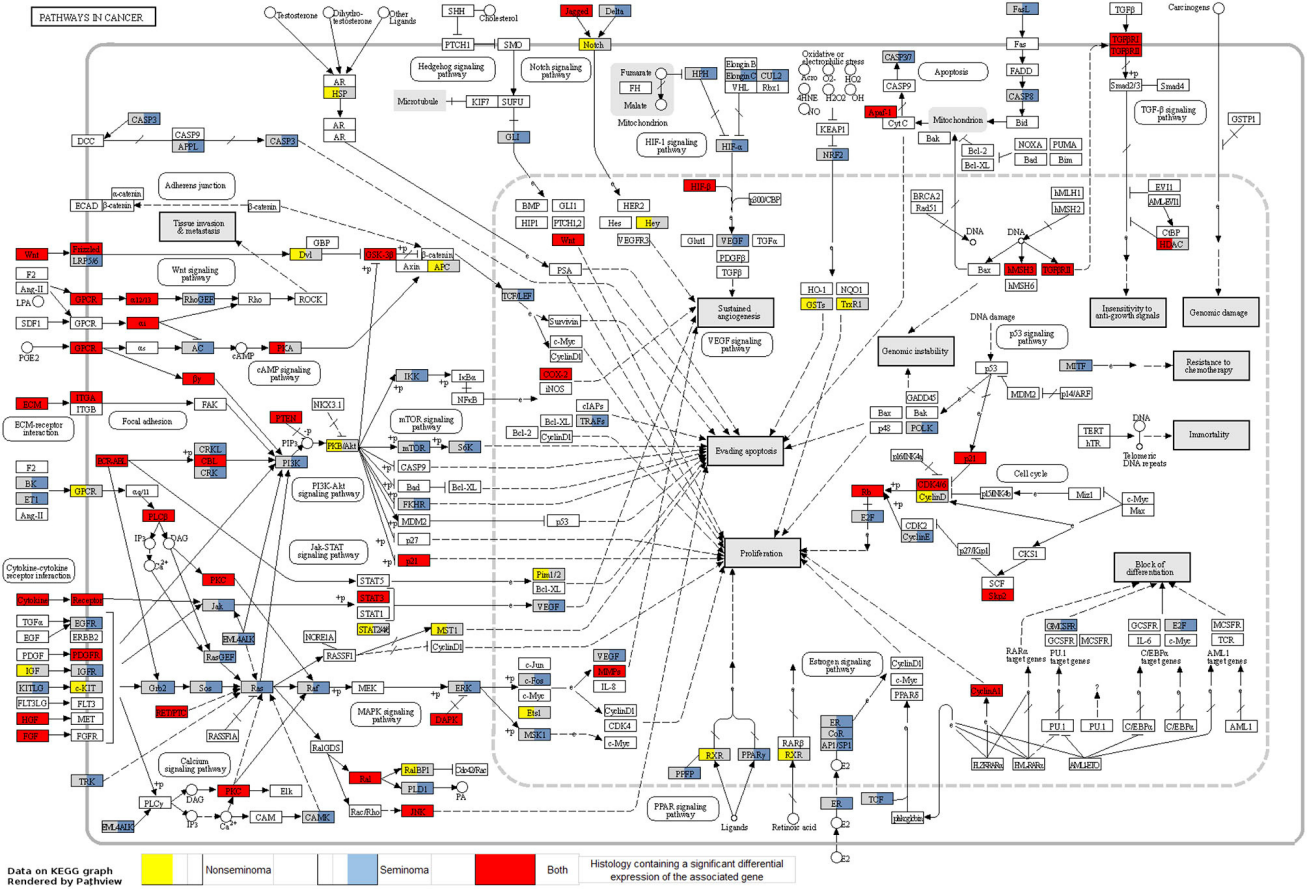
Map of the KEGG pathway “Pathways in Cancer” displaying genes with differentially expressed mRNA fragments and miRNA targets specific to histologies. The genes that are partially highlighted and color coded are significantly associated within seminoma (blue) or non-seminoma (yellow). Genes fully highlighted are significantly associated when both histologies (red) are included in the analyses.

**Figure 8:**
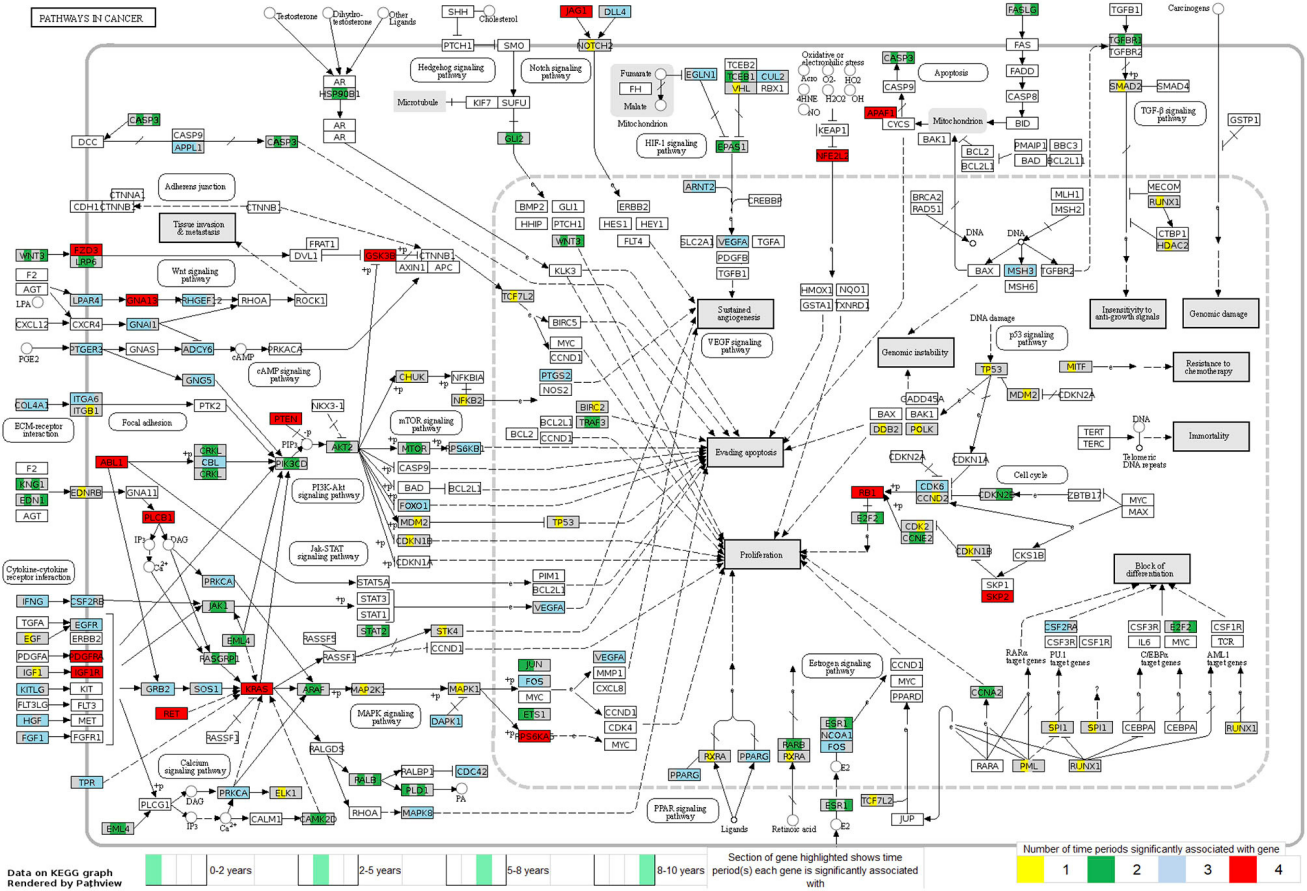
Map of the KEGG pathway “Pathways in Cancer” displaying genes with differentially expressed mRNA fragments and miRNA targets specific to a time period prior to diagnosis. The genes partially highlighted are significantly associated within the four time frames 0–2, 2–5, 5–8, or 8–10 years prior to diagnosis. Section of the gene highlighted corresponds to which time frame the gene is associated with, and color of the gene corresponds to how many time frames it can be found in.

Looking at each sub analysis, enrichment of miRNAs and mRNA fragments among the 88 significant RNAs in all TGCTs revealed several cancer related pathways, including mTOR, MAPK and ErbB2 (Figure 6). In seminomas the expressed miRNAs and mRNA fragments also showed the cancer related pathways mTOR, MAPK and AMPK as well as the proteoglycans in cancer pathway. Similar pathways were present in non-seminomas as well, with miRNAs and mRNA fragments showing cancer related pathways, with mTOR, MAPK, ERBb2, and PI3K-Akt showing highest significance (Figure 6). Finally, all four time frames showed significant pathways, including the mTOR signaling pathway which was one of the top 10 more significant pathways throughout, with the MAPK and Erb2 pathways also showing up once more within the time frames (Figure 6). Visualization of the pathway “Pathway in Cancer,” selected since it summarizes the most relevant cancer paths, showed us which of the associated genes were present throughout the time frames and across histologies (Figures 7, 8). These pathview plots show a notable difference between seminoma and non-seminoma. For example the KITLG-IGFR/EGFR-Grb2-Sos-Raf-Ras-ERK path is specific to seminomas. Of particular interest to us was the presence of the PTEN in the pathways, known from TGCT-genome-wide association studies, which were significantly associated with all four time periods and both histologies.

## Discussion

We found the RNA profiles in pre-diagnostic serum of TGCT patients to be different from that of controls, and observed that the difference was relatively stable across the pre-diagnostic time period of 10 years. Although previous studies have identified TGCT specific RNA profiles in patients (28–30), this has not been described for the pre-diagnostic period, as seen in our study. In contrast to our findings, highly dynamic pre-diagnostic RNA signals have been observed in lung cancer and breast cancer samples (27, 47, 48). The reason for this contrast may partly be due to the localization of the tumor within the testis. Typically, the developed GCNIS is found above the basement membrane in seminiferous tubules, which are located in the intratesticular environment and therefore separate from the bloodstream *via* the blood testis barrier (BTB) (49, 50). The BTB's primary function is to prevent both antibodies and T lymphocytes from affecting the testis (51). Furthermore, angiogenesis in breast and lung cancer is well-documented, showing a closer physical interaction between these tumors and circulatory system than what applies to TGCT (52, 53). GCNIS may also be less affected by the immune system (54, 55) as the BTB provides adequate protection against immune response during the early stages of the malignant growth. Another potential implication of the presence of the BTB function is a delay in serum signals. Changes in the GCNIS embedded in the seminiferous tubules do not necessarily appear in blood unless the tumor causes disruption of the BTB (56, 57). Further studies are needed to show how the interference of TGCT with the BTB affects the levels of circulating RNAs.

In this study we found that differentially expressed levels of mRNAs were related to both testis development and known cancer pathways, as well as the lncRNA, TSIX which exhibited increased levels in TGCT patients. The role of TSIX in X-chromosome inactivation (XCI) has been widely reported (58–60), acting as the antisense repressor of XCI. The expression of TSIX is limited to pluripotent cells and the testis. A zinc finger protein, GLI, binds to the 5′ ends of TSIX to reduce TSIX expression and block the initiation of XCI. Physiologically, XCI is observed during spermatogenesis, but can also be expressed in TGCTs through supernumerary X chromosome constitution (61). XCI has been observed in TGCT of different histogenesis and may be a potential biomarker for some types of TGCT (62).

Alongside increased levels of TSIX, we also observed a positive log_2_fc with RAB21, an oncogene involved in mediating endocytosis and connected to the tumorigenesis and vesicle transport mechanisms of the other Rab proteins (63). The silencing of RAB21 has also been utilized to induce apoptosis in glioma cells and can also be used to significantly inhibit cell growth (64). The increased presence of RAB21 in the serum of patients after diagnosis of TGCT has not been observed before, however, previous studies have elucidated the function of a similar RAS family associated protein, RAB12, in rat testis development, especially noting the high expression in Sertoli cells (SC) (65). RAB GTPases have been identified as mediators for vesicle trafficking in cancer, including both RAB21 and RAB12 (63).

Investigating of the different histology patterns showed that despite the increased positive log_2_fc in non-seminomas, there are still RNA signals, such as mRNA TEX101, that are independent of the histology. TEX101 is involved in the production of spermatozoa, and studies using animal models have shown that males with a disrupted TEX101 gene often produce spermatozoa that are unable to fertilize, despite looking normal (66). Previous studies have found links between TGCT and changes in fertility (67). These findings include men with TGCT having fewer children than average, as well as a lower proportion of those children being male. Other studies have found that abnormal semen characteristics can be observed in men who later develop TGCT, indicating that these two aspects are aetiologically linked (68).

As hypothesized previously, the functions of the mRNAs identified as stable across all time frames could give some indication as to how GCNIS develops into tumors. Fragments of the mRNA for AP000295.1 had lower levels in cases compared to controls. AP000295.1 mRNA expression has been previously noted in the Human Protein Atlas (HPA) project, and HPA shows RNA-seq tissue data with high protein-coding transcripts per million (pTPM) counts of AP000295.1 in the blood constituents, granulocytes and monocytes (69). AP000295.1 belongs to a network of genes associated with kinase binding and type I interferon binding, AP000295.1 is also a paralog of the gene IFNAR2 with 100% match between target and query genes as seen using Ensembl (release 99) (70). Of note here is that IFNAR2 has been detected on the surface of pre-sertoli cells (pSC) (71). Previous studies have demonstrated the importance of Sertoli cells in the BTB formation and homeostasis (72), thus potentially explaining the presence of an RNA signal in serum despite the impermeable BTB. Through the reduction in expression of IFNAR2 in pSC's surface, inadequate formation and maintenance of the BTB could occur, causing a breakdown of this filter between the testis and the blood, thus explaining higher presence of signals of tumor development in serum.

Circulating miRNAs have been identified as both prognostic and diagnostic markers in biliary tract cancers (BTC), and this has led to earlier diagnosis and less invasive procedure of BTC than previously used techniques (25). The circulating miRNAs used for BTC diagnosis were reported to be stable in serum and showed significantly different miRNA expression in patients with BTC and controls, and there also may be ethnic differences. Circulating biomarkers has also been identified in colorectal cancer (CRC), specifically the exosomal miRNAs, miR-150-5p, and miR-99b-5p were found to be downregulated in CRC patients compared to healthy individuals (26, 73). This demonstrates the potential for their later use in clinical settings as a non-invasive diagnostic technique.

Pathways enrichment analysis showed overall many associated cancer related pathways across the 10-years period prior to TGCT diagnosis. The time frames 2–5 and 5–8 years prior to diagnosis showed the highest numbers of enriched pathways, many of which were cancer related. A possible explanation for the absence of enriched pathways in the 0–2 year time interval, could be an overall increase of all RNA types in serum as the tumor develops, this would effectively mask cancer pathways from being detected due to the large amounts of noise from aberrant RNA expression.

The presence of PTEN and KRAS genes in our pathway maps (Figures 7, 8), shows that these genes have been significantly differentially expressed in the prediagnostic cases over controls and can be found in all four time frames and both histologies. PTEN is a tumor suppressor gene that has been previously found to play an important role in the progression of intratubular germ cell neoplasias into the more mature invasive germ cell tumors. PTEN is often highly expressed in normal germ cells, but it is absent in the most common forms of TGCT (74).

Genome-wide association studies for TGCT have consistently shown that KITLG (ligand for the receptor tyrosine kinase) is implicated in TGCT susceptibility and with high effect sizes (75, 76). In these studies, the marker genotypes in KITLG were associated with both seminoma and non-seminoma TGCT without indication that genotype associations differed between the two subtypes. Our data thus suggest that epigenetics might play a more important role in the heterogeneity between seminoma and non-seminoma than hitherto recognized. More specifically, our results fit nicely with the TGCT histology specific DNA methylation patterns for KIT, shown by Shen et al. (77). Shen et al. (77) showed histology specific DNA methylation patterns for KIT and we have now shown differential RNA expression of this pathway to be specific to seminomas. Together this indicates histology-specific epigenetic mechanisms at play in the KIT–TGCT association.

A strength of this study is the large and robust RNA dataset. To our knowledge this is the largest study of the RNA profiles from TGCT patients prior to diagnosis also including harmonized confounders collected from health surveys (35). The time interval throughout which the samples were collected is also a strength of this study. With up to 10 years time before diagnosis we were able to observe TGCT development and elucidate the body's response.

The sample size is still a limitation, and stratified analyses on histology and time to diagnosis could benefit from an increase in statistical power. Serum samples and survey data were collected over a period of more than 30 years, and lifestyle factors such as smoking and BMI have changed within this time period as well as carcinogens in the environment, such as organochlorine pesticides (78–80). Cases and controls were matched based on year of donation/storage time and sampling protocol, to minimize the possible bias of temporal/secular trends of exposure variables that could have biased the study.

Primarily, the new insights into TGCT carcinogenesis helps us better understand the development of the disease, which is thought to be initiated *in utero*. Stable serum RNAs in the pre-diagnostic period have the potential to be biomarkers for earlier detection. Early diagnosis of TGCT would lead to less use of cisplatin treatment, and thereby reduce long-term adverse effects, such as risk of second primary cancer and cardiovascular disease. Hazard ratios for all secondary cancers after a single cisplatin-based chemotherapy cycle were significantly lower than hazard ratios after two or more cycles (81).

In conclusion, RNA levels associated with cancer related pathways were different between individuals who developed TGCT, compared to matched controls. There is some loss of pathway signals closer to diagnosis, however, there is inherent reduced levels of serum RNAs in cases compared to controls for most of the 10 years pre-diagnostic follow-up time. The presence of these stable RNA signals may help in the identification of biomarkers give insights to molecular mechanisms driving TGCT development.

## Data Availability Statement

The datasets generated for this article are not readily available because of the principles and conditions set out in articles 6 (1) (e) and 9 (2) (j) of the General Data Protection Regulation (GDPR). National legal basis as per the Regulations on population-based health surveys and ethical approval from the Norwegian Regional Committee for Medical and Health Research Ethics (REC) is also required. Requests to access the datasets should be directed to the corresponding authors.

## Ethics Statement

The studies involving human participants were reviewed and approved by Norwegian regional committee for medical and health research ethics. The patients/participants provided their written informed consent to participate in this study.

## Author Contributions

TH and TR designed the study. JB and SU performed the analyses of the data. JB, TH, and TR drafted the manuscript. All authors discussed the results, contributed to the writing, and approved the final manuscript.

## Conflict of Interest

The authors declare that the research was conducted in the absence of any commercial or financial relationships that could be construed as a potential conflict of interest.
