# Supplementary material for: Serum RNA Profiling in the 10-Years Period Prior to Diagnosis of Testicular Germ Cell Tumor
Source: Front Oncol. 2020 Oct 28;10:574977. doi: 10.3389/fonc.2020.574977 (PMC7673397; doi:10.3389/fonc.2020.574977)
Supplement: Supplementary file 1 [file Data_Sheet_1.PDF]

Table S1. Testicular germ cell tumour cases and controls descriptives. 65 samples included were from red cross blood donors (RCBD) and 125 were from donors participating in health examinations (HEBD), in total 190.

	Non-seminoma subtypes	Cases	Controls
**Age at sampling**, years			
Mean (SD)		34.84 (6.49)	35.48 (6.69)
**Time to diagnosis**, years			
Mean (SD)		4.52 (3.16)	-
**Subtype**			
seminoma		52	-
non-seminoma		27	-
	Mixed tumour of seminoma and non-seminoma component	13	
	Embryonic carcinoma	7	
	Teratoma, embryonic teratoma, immature teratoma, malignant teratoblastoma, carcinoma of teratoma	4	
	Mixed germinal cell tumour UNS (non-seminoma components only)	3	
**Blood donor group**			
1 - HEBD from 1972–1978, iodoactetate added		5	12
2 - HEBD from 1979–1986		15	23
3 - HEBD from 1987–2004 collected in separating gel tubes		34	36
4 - RCBD from 1973–1979		12	19
5 - RCBD from 1980–1990		9	17
6 - RCBD from 1997–2004		4	4
**Total**		79	111

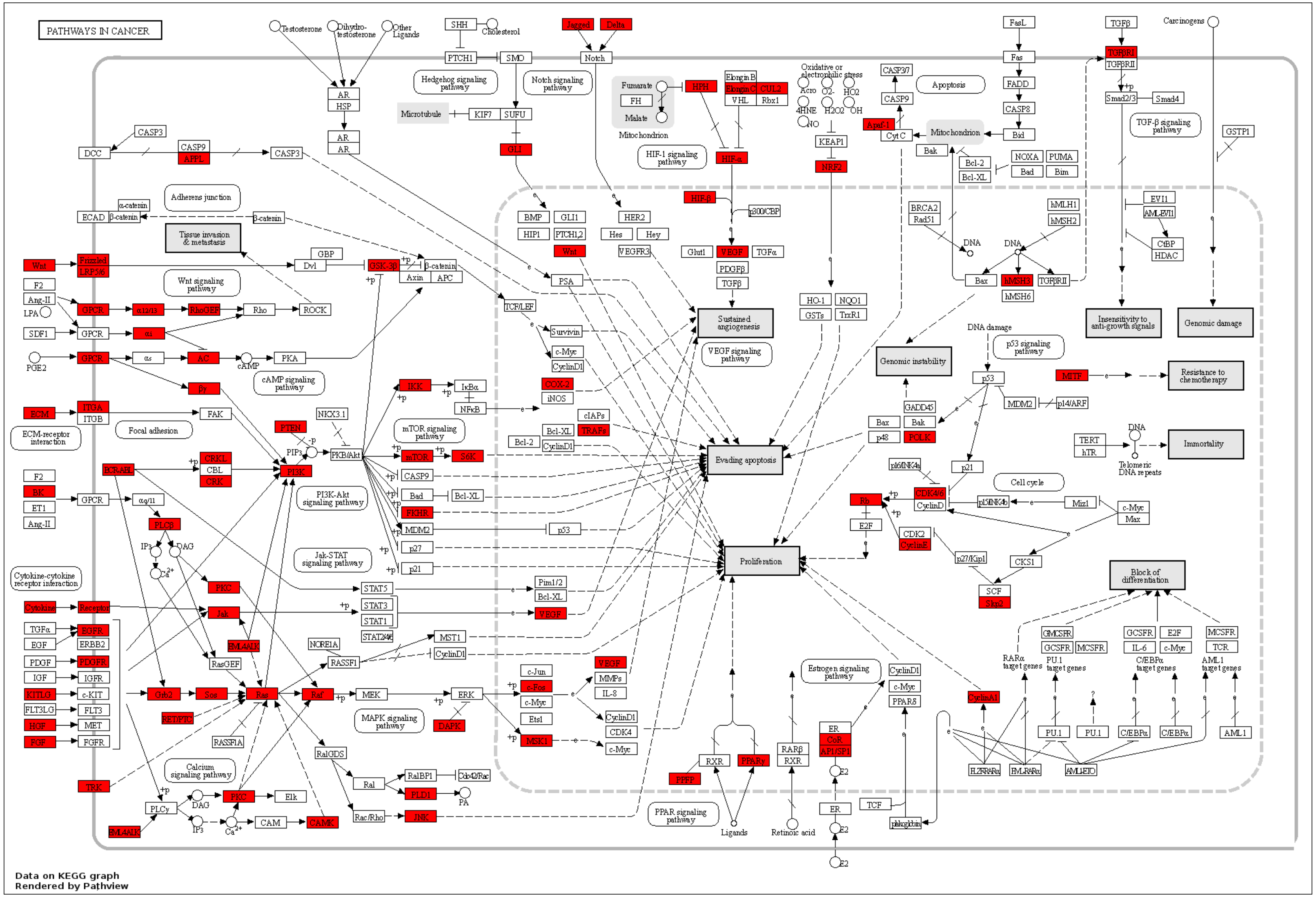

Figure S1. Map of the KEGG pathway "Pathways in Cancer", genes highlighted are significantly associated with genes in our all cases vs all controls differential gene expression analysis.
